# Epstein-Barr Virus-Positive Diffuse Large B-Cell Lymphoma in the Elderly: A Matched Case-Control Analysis

**DOI:** 10.1371/journal.pone.0133973

**Published:** 2015-07-29

**Authors:** Chen-Ge Song, Jia-Jia Huang, Ya-Jun Li, Yi Xia, Yu Wang, Xi-Wen Bi, Wen-Qi Jiang, Hui-Qiang Huang, Tong-Yu Lin, Zhi-Ming Li

**Affiliations:** 1 Sun Yat-sen University Cancer Center, State Key Laboratory of Oncology in South China, Collaborative Innovation Center for Cancer Medicine, Guangzhou, Guangdong, China; 2 Tumor Hospital of Hunan Province, Changsha, Hunan, China; United Arab Emirates University, UNITED ARAB EMIRATES

## Abstract

**Background:**

Epstein-Barr virus (EBV)-positive diffuse large B-cell lymphoma (DLBCL) in the elderly has rarely been reported. This study aimed to explore the clinical characteristics and prognosis of this entity.

**Methods:**

In situ hybridization (ISH) analysis of Epstein-Barr virus (EBV) and immunohistochemistry was performed in 230 tumor specimens from consecutive de novo DLBCL patients over 50 years old. A matched-case control analysis (1:3) was utilized to compare EBV-positive and EBV-negative DLBCL in the elderly.

**Results:**

A total of 16 patients (7.0%) were diagnosed with EBV-positive DLBCL. Of these 16 cases, the median age was 62 years, with a male to female ratio of 11:5. Elderly EBV-positive DLBCL patients had a higher incidence of non-germinal center B-cell (non-GCB) subtypes (87.5%) and high Ki67 (75%) and CD30 expression (93.8%). For EBV-positive patients undergoing initial chemotherapy, 7 of 16 (43.8%) had complete remission, 2 (12.5%) had partial remission, 2 (12.5%) had stable disease, and 5 (31.3%) had progressive disease. The median overall survival was 9 months for the EBV-positive patients. A matched-case control analysis suggested that EBV-positive patients had inferior survival outcomes compared with EBV-negative patients (3-year progression-free survival [PFS]: 25% vs. 76.7%, respectively; 3-year overall survival [OS]: 25% vs. 77.4%, respectively; P<0.001).

**Conclusion:**

EBV-positive DLBCL of the elderly is associated with an inferior clinical course and inferior survival outcomes. The role of EBV in this disease and the optimal management of this subgroup warrants further investigation.

## Introduction

Diffuse large B-cell lymphoma (DLBCL) is the most common subtype of B-cell non-Hodgkin lymphoma (B-NHL). With advances in diagnosis and treatment, the outcome of this malignancy has improved. However, for DLBCL of the elderly, there is still a need for improvement in terms of prognosis. Among elderly patients with DLBCL, we focused on a unique subtype, the Epstein-Barr virus-positive DLBCL. Based on the WHO 2008 classification, this clinical entity is defined by age at diagnosis over 50 years, no secondary immune deficiencies, and detectable EBV infection within the tumor cells [1]. Though first reported in 2003 [2,3] and identified as a subtype of DLBCL in 2008, this disease is not universally recognized and remains controversial for both clinicians and pathologists. This malignancy has a low incidence rate, reportedly ranging from 3.5% to 9% of all cases of DLBCL among the elderly [4,5,6,7]. Because there is no clearly superior treatment for this disease, some lymphoma specialists do not feel the need to distinguish this subtype from “normal” senile DLBCL. These factors may explain the lack of existing literature focusing on this condition.

To contribute to a better understanding of this disease, we conducted a retrospective analysis and a matched case-control study between EBV-positive and EBV-negative cases. Clinicopathological features and treatment outcomes were compared to shed light on the unique features of this disease.

## Materials and Methods

### Patients

A total of 230 consecutive patients over 50 years of age at the time of diagnosis with de novo DLBCL were retrospectively reviewed in this study. All of the patients included in this series fulfilled the following criteria: (1) pathologically confirmed diagnosis of DLBCL according to the WHO Classification of Tumors of Haematopoietic and Lymphoid Tissues [1]; (2) age older than 50 years; (3) EBV in situ hybridization had been performed; (4) no previous treatment; (5) no previous malignancy or second malignancy; and (6) clinical data and follow-up information available. Patients with human immunodeficiency virus (HIV) infection were excluded. The cases were diagnosed by experienced hematopathologists at Sun Yat-Sen University Cancer Center between January 2001 and December 2011. Sixteen patients were diagnosed with elderly EBV-positive DLBCL based on the WHO Classification of Tumors of Haematopoietic and Lymphoid Tissues [1]. All 230 patients received treatment at the Sun Yat-Sen University Cancer Center. This study was approved by the Institutional Review Board of Sun Yat-Sen University Cancer Center. Participants provided their written informed consent to participate in this study. The ethics committee approved this consent procedure.

### Immunohistochemical staining and analysis

Immunohistochemical (IHC) staining and analysis was carried out using the following antigens: CD20 (L26, 1:200), CD79a (1:50), CD45 (LCA, 1:20), CD3 (1:200), CD5 (1:100), CD10 (1:50), BCL-6 (1:10), MUM-1 (1:50), BCL-2 (1:80), Ki-67 (1:100), CD30 (1:20), CD38 (1:10), CD138 (1:50), UCHL-1 (CD45RO, 1:200), κ (1:300), λ (1:400), OCT-2 (1:500), BOB-1 (1:500), cyclin D1 (1:50), ALK (1:10), CD43 (1:320), PAX-5, and Vs38c (P63, 1:10) (DakoGlostrup, Denmark). The routine immunohistochemistry method was carried out as described in detail previously [8]. Germinal center B-cell-like (GCB) and non-GCB groups were subclassified according to the algorithm of Hans [9]. IgH/C-Myc and IgH/Bcl-2 translocation was detected using fluorescent in situ hybridization (FISH) analysis in selected cases [10].

### EBV infection detection

Based on the manufacturer’s instructions, in situ hybridization (ISH) analysis for EBV-encoded small RNAs (EBERs) was carried out on paraffin sections with fluorescein-conjugated peptide nucleic acid probes (Dako).

### Matched case-control study design

Patients who received treatment during the course of their disease were selected from the remaining 214 EBV-negative patients with de novo DLBCL diagnosed at Sun Yat-Sen University Cancer Center between January 2001 and December 2011 to serve as matched controls for patients with EBV-positive DLBCL.

The control procedure employed strict matching criteria: standard international prognosis index (IPI) score (age, Eastern Cooperative Oncology Group Performance Status [ECOG PS], lactate dehydrogenase [LDH] level, Ann Arbor stage, and number of extranodal sites); gender (male or female); age (±5 years); treatment regimen (including rituximab addition); Ki67 index (≥80% or <80%); Bcl-2 status; and GCB/non-GCB cell origin. All of these factors were fully matched between the study case and the three controls. If more than three controls were matched with a case, three control cases were picked randomly to complete the matching.

### Statistical methods

Progression-free survival (PFS) was measured from the date of diagnosis to the date of first relapse, progression, death, or last follow-up. Overall survival (OS) was defined as the time from diagnosis to death or last follow-up. The survival curve was constructed by the Kaplan-Meier method, and comparisons between groups were analyzed by the log-rank test and Bonferroni correction. The clinicopathological variables of the two groups were analyzed using a Pearson’s χ^2^ test for categorical variables and the Mann-Whitney test for continuous variables. A two-tailed P-value <0.05 by log-rank test was considered statistically significant. Statistical analysis was performed using the Statistical Package for Social Sciences (SPSS) 16.0 statistical software package (SPSS, Inc., Chicago, IL, USA).

## Results and Discussion

### Clinical features of patients

Clinical data for 230 consecutive elderly (over 50 years) patients with de novo DLBCL were collected in this study. A total of 16 patients (7.0%) were diagnosed with EBV-positive DLBCL of the elderly. All 16 patients were HIV-negative and had no history of other lymphoproliferative disorders, organ/hematopoietic stem cell transplantation, or congenital immune deficiencies. The median age of these 16 patients was 62 years (range, 51–76 years), and the male-to-female ratio was 11:5. Eight patients (50%) had stage I/II disease, and 8 patients (50%) had stage III/IV disease according to the Ann Arbor staging system. B symptoms were present in 8 cases (50%). Bulky disease (mass ≥7 cm) was not observed in any of the cases. The serum lactate dehydrogenase (LDH) level was elevated in 7 cases (43.8%). Seven patients (43.8%) had extranodal involvement. One patient (6.3%) had a low IPI score (0–1 risk factors), 4 patients (25%) had low-intermediate IPI scores (2 risk factors), 7 patients (43.7%) had high-intermediate IPI scores (3 risk factors), and 4 patients (25%) had high-risk IPI scores (4–5 risk factors). One patient (6.3%) had bone marrow involvement. The PS was <2 in 7 patients (43.7%). Differences in clinical characteristics between EBV-positive and EBV-negative DLBCL patients are summarized in Table 1. As expected from the matching method, there were no statistically significant differences in the main clinical characteristics between patients in the study and control groups, as shown in Table 2; the only exception was the ECOG PS score. EBV-positive patients in the study group tended to have poorer performance scores than the control group. Detailed clinical information for each EBV-positive patient is listed in Table 3.

**Table 1:** Characteristics of EBV-positive and EBV-negative elderly DLBCL patients.

		Total	EBV positive(n = 16)	EBV negative (n = 214)	P value
Gender	Male	147	11	136	0.676
	Female	83	5	78	
Age	<60	121	5	116	0.076
	>=60	109	11	98	
IPI	<=2	173	6	167	<0.001
	>2	57	10	47	
aaIPI	<2	181	3	178	<0.001
	>=2	48	12	36	
Ki67 expression	<80%	119	4	115	0.026
	>=80%	111	12	99	
Cell Origin	GCB	81	2	79	0.049
	Non-GCB	149	14	135	
Bcl2 expression	Positive	121	10	111	0.411
	Negative	109	6	103	
CD30 expression	Positive	83	15	68	<0.001
	Negative	147	1	146	
LDH status	elevated	112	5	107	0.148
	normal	118	11	107	
Extranodal sites	0 or 1	193	15	178	0.588
	2 or more	38	2	36	
ECOG status	0 or 1	195	7	188	<0.001
	2 or higher	35	9	26	
Ann Arbor Stage	I-II	82	8	74	0.214

EBV, Epstein-barr Virus; IPI, International Prognosis Index; aaIPI, age-adjusted international prognosis Index; GCB, Germinal center B cell; non-GCB, non Germinal center B cell; Bcl-2, B cell lymphoma -2; LDH, lactate dehydrogenase; ECOG, Eastern cooperative oncology group.

**Table 2:** Comparison of EBV-positive (study group) and EBV-negative (control group) elderly DLBCL patients in a matched case-control analysis.

		Total	EBV positive(n = 16)	EBV negative (n = 48)	P value
Gender	Male	44	11	33	1
	Female	20	5	15	
Age	<60	20	5	15	1
	>=60	44	11	33	
IPI	Low to medium <=2	24	6	18	1
	Medium to high >2	40	10	30	
aaIPI	Low to medium <2	28	7	21	1
	Medium to high >=2	36	9	27	
Ki67 index	<80%	16	4	12	1
	>=80%	48	12	36	
Cell Origin	GCB	8	2	6	1
	Non GCB	56	14	42	
Bcl2	Positive	40	10	30	1
	Negative	24	6	18	
CD30	Positive	23	15	8	<0.001
	Negative	41	1	40	
LDH status	Elevated	23	5	18	0.66
	Normal	41	11	30	
Extranodal sites	0 or 1	49	14	35	0.317
	2 or more	15	2	13	
ECOG status	0 or 1	50	7	43	<0.001
	2 or higher	14	9	5	
AnnArbor Staging	I,II	30	8	22	0.77
	III,IV	34	8	26	
Regimen of chemotherapy	CHOP	56	14	42	1
	EPOCH	8	2	6	
Rituximab Addition	Yes	32	8	24	1
	No	32	8	24	
Radiotherapy	Yes	0	0	0	
	No	64	16	48	

EBV, Epstein-barr Virus; IPI, International Prognosis Index; aaIPI, age-adjusted international prognosis Index; GCB, Germinal center B cell; non-GCB, non Germinal center B cell; Bcl-2, B cell lymphoma -2; LDH, lactate dehydrogenase; ECOG, Eastern cooperative oncology group; CHOP, cyclophosphamide, doxorubicin (may be substituted by epirubicin or peglyated liposome doxorubicin), vincristine,prednisone; EPOCH, etoposide, cyclophosphamide, doxorubicin, vincristine, prednisone.

**Table 3:** Clinical features and survival outcomes of 16 EBV-positive elderly DLBCL patients.

Patient Number	Age/Gender	Site of involvement	Stage	IPI	Initial Chemotherapy and response	Outcome	Survival (months)
1	51/F	LN	IIA	2	CHOP×4 CR	DOD	12
2	76/F	LN	IIB	3	CHOP×3 CR	DOD	6
3	59/M	LN, Left Tonsil	IIIA	3	CHOP×5 CR	AND	49
4	62/F	LN, Left Lung	IVEB	4	EPOCH×4 SD	AWD	22
5	62/M	LN, bilateral Hip	IVEB	4	CHOP×3 PR	DOD	8
6	62/M	LN, Stomach	IVEA	3	CHOP×2 PD	DOD	2
7	67/F	LN,BM,Right Kidney	IVEB	5	CHOP×6 PD	DOD	10
8	55/M	LN	IIA	3	R-CHOP×2 PD	DOD	1
9	55/M	LN, Colon	IVEB	3	CHOP×2 SD	DOD	5
10	75/M	LN	IIB	2	R-CHOP×4 CR	AND	44
11	60/M	LN	IIA	1	R-EPOCH×5 CR	AND	49
12	74/M	LN, Left Kidney	IVEB	3	R-CHOP×4 PR	DOD	8
13	64/M	Spleen	ISA	3	R-CHOP×6 CR	AND	39
14	56/M	LN	IIA	2	R-EPOCH×4 CR	AND	60
15	75/F	LN, Stomach	IVEB	4	R-CHOP×4 PD	DOD	7
16	51/M	LN	IIA	2	R-CHOP×2 PD	DOD	7

M, male; F, female; LN, lymph node; IPI, International Prognostic Index; R, Rituximab; CHOP, cyclophosphamide, doxorubicin(may be substituted by epirubicin or peglyated liposome doxorubicin), vincristine,prednisone; EPOCH, etoposide, cyclophosphamide, doxorubicin, vincristine, prednisone; CR, complete remission; PR, partial remission; SD, stable disease; PD, progressive disease; AWD, alive with disease; AND, alive with no evidence of disease; DOD, died of disease.

### Immunohistochemical studies

Among the 16 cases with EBV-positive DLBCL, 14 cases (87.5%) were categorized as non-GCB type, while 2 of 16 (12.5%) were categorized as GCB-type. Ki-67 was immune-labeled to identify a high proliferation index (≥80%) for lymphoma cells in 12 of 16 (75%) cases. No case was positive for FISH detection of immunoglobulin heavy chain (IGH)/C-myc rearrangement. The main pathologic characteristics of the study group and control group are listed in Table 2.

### Response and survival analysis

**Fig 1:**
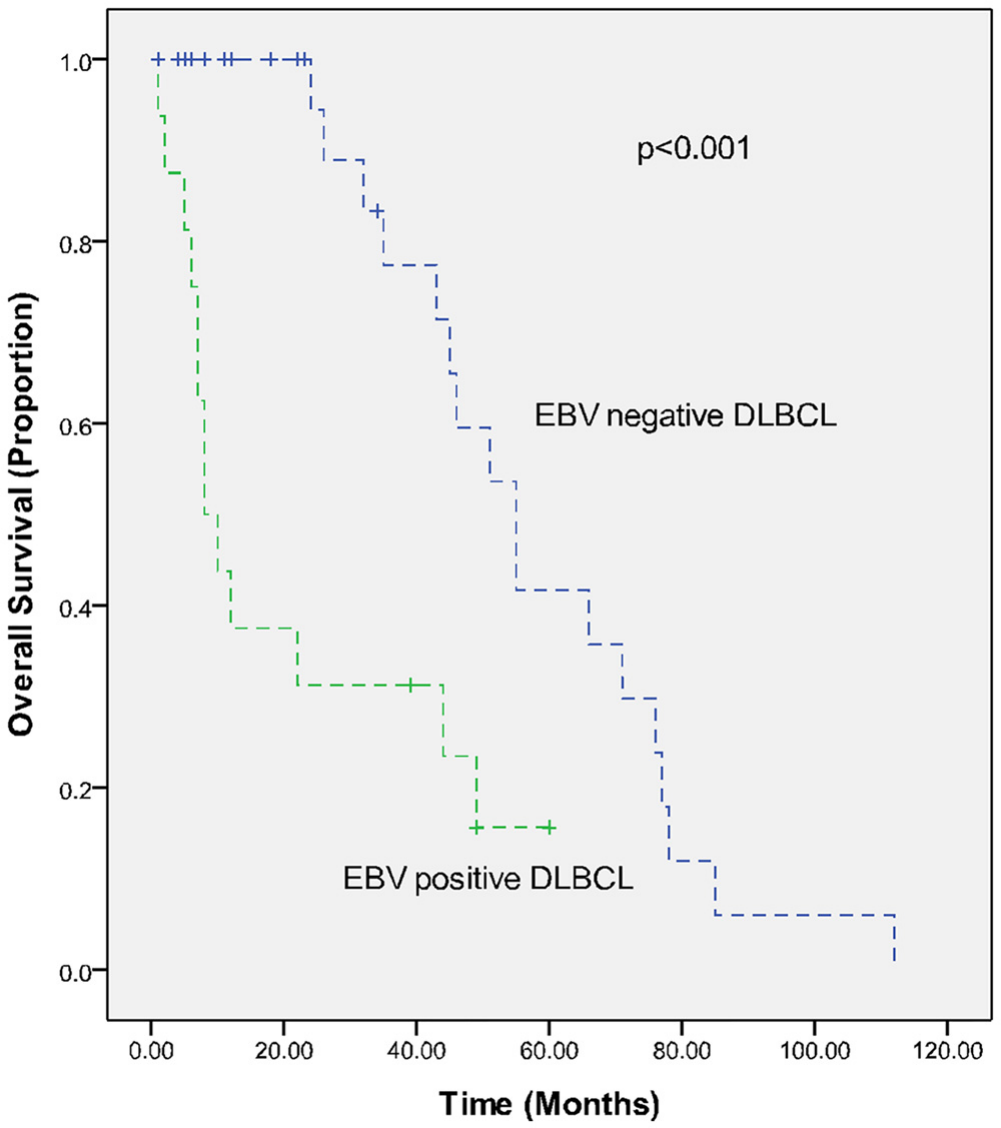
Overall survival (OS) in EBV-positive (study group) and EBV-negative (control group) elderly DLBCL patients in a matched case-control analysis.

**Fig 2:**
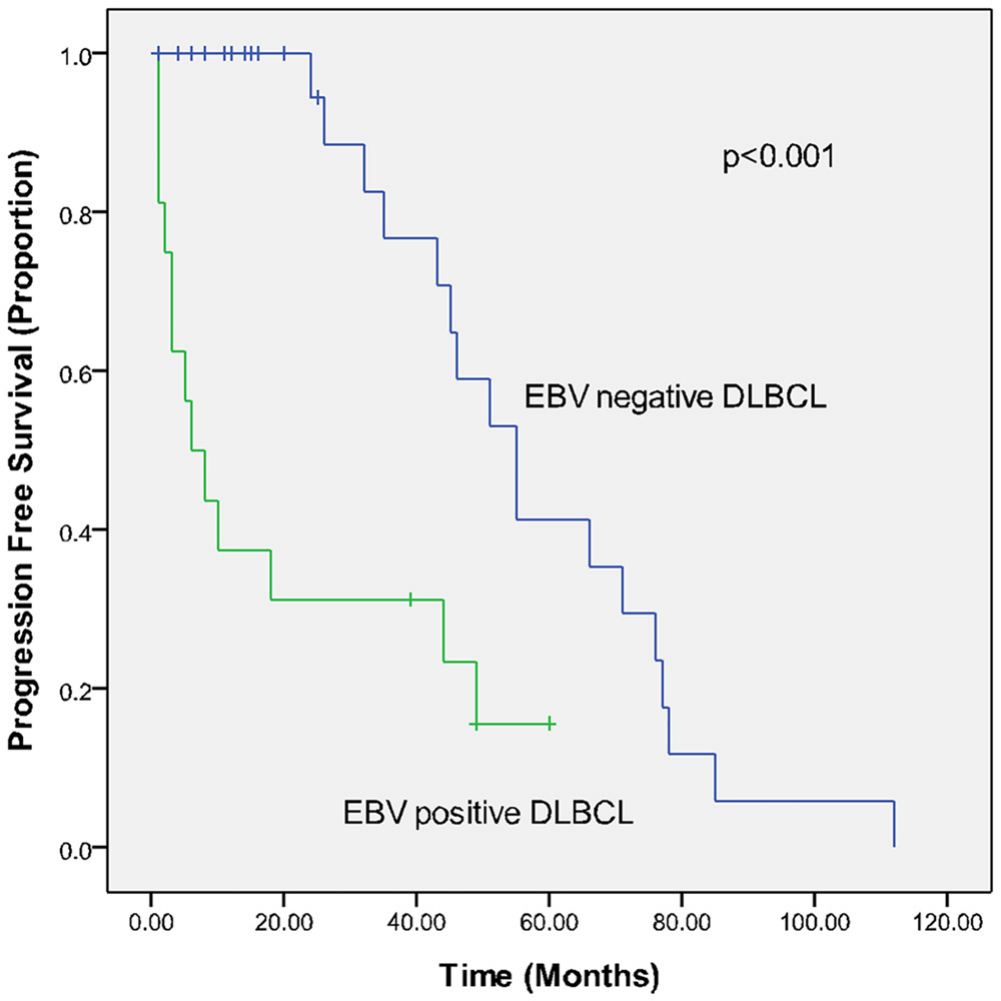
Progression-free survival (PFS) in EBV-positive (study group) and EBV-negative (control group) elderly DLBCL patients in a matched case-control analysis.

The treatments of the 16 EBV-positive de novo DLBCL cases are summarized in Table 3. All of the patients with EBV-positive DLBCL in this series received CHOP (cyclophosphamide, doxorubicin, vincristine, and prednisone) or EPOCH (etoposide, prednisone, vincristine, cyclophosphamide, doxorubicin) as first-line chemotherapy, and 8 patients received rituximab in addition to chemotherapy. No patient received curative radiotherapy pre- or post-chemotherapy, nor did any patient receive autologous stem cell transplantation (SCT) during the course of his or her disease. Following initial therapy, 7 of 16 (43.8%) cases achieved complete remission, 2 (12.5%) achieved partial remission, 2 (12.5%) had stable disease, and 5 (31.3%) had progressive disease. At the time of analysis, 14 patients (87.5%) had died; all of the deaths were due to lymphoma. The median OS time was 9 months. The 3-year PFS and OS rates were each 25%. Based on univariate analysis, the variables associated with a longer OS included the following: PS ≤1 (P = 0.033), extranodal involvement at <2 sites (P = 0.007), age-adjusted IPI (aaIPI) <2 (P = 0.001), IPI score <2 (P = 0.032), normal LDH (P = 0.002), and complete remission (CR) following initial therapy (P = 0.013). As shown in Table 4, the CR rate in the control group was significantly higher than in the study group (77.1% vs. 43.8%, P = 0.013). With a median follow-up of 47 months (range 1–121 months), the PFS and OS in the EBV-positive DLBCL group were significantly poorer than the EBV-negative DLBCL group, as shown in Figs 1 and 2 (3-year PFS: 25% vs. 76.7%; 3-year OS: 25% vs. 77.4%; P<0.001 for both PFS and OS).

**Table 4:** Treatment responses and survival outcomes of EBV-positive (study group) and EBV-negative (control group) elderly DLBCL patients.

		EBV positive (n = 16)	EBV negative (n = 48)	P value
Response	CR	7	37	0.013
	No CR	9	11	
Survival	3 Year PFS	25%	76.7%	<0.001
	3 Year OS	25%	77.4%	<0.001

CR, complete remission; PFS, progression free survival; OS, overall survival.

## Discussion

EBV-positive DLBCL is very rare among DLBCL patients over 50 years old. In this series, only 7% of elderly DLBCL patients found to be EBV-positive. As the available literature indicates, the incidence is relatively low [4,5,6,7]. As a result, for most single-center studies, the sample size is limited. Earlier literature revealed the adverse prognostic factors associated with this condition [4,5,6,7]. This trend, however, was not observed in studies based on Western populations [7]. Consistent with earlier studies of Eastern populations, we also found that the prognosis of this disease was significantly poorer than its EBV-negative counterpart[4,5,6]. EBV-positive cases appeared to be associated with more adverse clinical parameters than EBV-negative cases. The question remains whether we can attribute the poor prognosis to these factors or to the unique characteristics of the disease, specifically the EBV infection.

EBV is one of the earliest viruses to have been associated with malignant tumors, including several types of leukemia, lymphoma and solid tumors [5]. Earlier studies demonstrated that this pathogen played an important role in carcinogenesis of lymphoma [5]. EBV latent infection usually targets B cell proliferation [5]. No study in the literature has focused on serological virus parameters such as EBV antibody levels and EBV DNA copy number or has shown that no significantly elevated serological EBV parameters could be detected. Except for EBER-positive cases, an IHC test can often find tumor cell membrane LMP1 expression, which is associated with chronic and latent EBV infection [11]. EBNA2 expression, which results in the transactivation of Myc, can also be detected [11]. To balance the population and demonstrate the prognostic value of EBV infection, a matched-case control study was performed to investigate whether differences existed.

EBV-positive DLBCL of the elderly carries many unfavorable clinical prognostic factors, with IPI being the most classic. IPI has been shown to be significant in many aggressive lymphomas [12,13]. As demonstrated by several large-scale randomized clinical trials, such as GELA 98.5 and RICOVER-60, higher IPI scores predict limited benefit from anthracycline-based chemotherapy, even when combined with rituximab [12,13]. Even after controlling for gender, age, and IPI score, the treatment response and survival outcomes for EBV-positive cases are still worse than for EBV-negative ones. In univariate analysis, age did not affect the survival; this result can be explained by the overall high age of the population. As a result, the IPI cannot explain all of the prognostic differences between EBV-positive and EBV-negative cases.

In regard to the adverse pathological prognostic factors, we mainly focused on cell origin and Bcl-2 expression. GCB/non-GCB subtype is an updated prognostic factor for DLBCL[14]. Non-GCB subtype patients have worse responses to CHOP-like regimens[15]. The non-GCB subtype makes up such a large proportion of elderly EBV-positive DLBCL that this malignancy is defined in some of the literature as a type of non-GCB DLBCL with nuclear factor (NF)-κB pathway activation [11]. However, when we focused on the EBV-positive cases, the expression of Bcl-2 did not show differences in survival. Bcl-2 expression is more often detected in EBV-positive cases. This phenomenon is also observed in Bcl-2/c-Myc co-expression DLBCL (double-hit lymphoma, DHL). DHL itself has a very poor prognosis, and, among these patients, even the IPI is of no significance for predicting the outcome [16]. These parameters can distinguish “worse” cases from “better” ones, but it is unable to recognize the “worst” cases. CD30 expression is also more often detected in EBV-positive cases. CD30 may be a potential target for novel agents such as brentuximab vedotin [17, 18], but is of limited prognostic value in DLBCL.

With limited sample sizes, prognostic factors may be of limited significance to clinicians. However, we also found that an elevated CRP level may predict worse survival, though the difference was not statistically significant; however, a similar trend has been noted in extranodal natural-killer cell/ T-cell lymphoma (ENK/TCL) [19]. These two malignancies both have abundant inflammatory cells infiltrating the background on histological slides [11]. The interactions among EBV, background inflammatory cells, and malignant cells remain unclear.

The results of our study suggest that EBV may play a role in the poor prognosis of EBV-positive DLBCL in the elderly. With the clinical and pathological parameters controlled and the population balanced, EBV-positive DLBCL in the elderly still has a worse prognosis than EBV-negative cases.

For a disease curable by chemotherapy, the first-line treatment regimen is of vital importance. Intensifying the treatment is one way to improve the prognosis; some intensified regimens, such as doxorubicin, cyclophosphamide, vindesine, bleomycin and prednisone (ACVBP), have shown limited advantages in either response rate or survival in normal senile DLBCL [20]. The advantages of EPOCH over CHOP appear to be significant in EBV-positive cases, but with a conclusion drawn from only 3 cases among 16, the external validity of this finding may be low. However, the toxicity for CHOP-like regimens is already serious; thus, intensifying the therapy regimen may not be a better option. A large proportion of non-GCB cell-origin cases may be targeted by novel agents, such as the Bruton’s tyrosine kinase (BTK) inhibitor ibrutinib [21,22]. Bortezomib, which is already a treatment option for non-GCB DLBCL, may also influence the NF-κB pathway[23,24]. The toxicity associated with this treatment is more serious in elderly patients than in younger patients. Grade III/IV hematological toxicity occurred in most EBV-positive DLBCL patients, but with adequate supportive care, recoverable neutropenia or thrombocytopenia is not as fatal as it once was. Anthracycline-associated cardiac toxicity is another concern, but with pre-treatment cardiac function testing such as ultrasonic cardiogram, multiple gated acquisition (MUGA) or electrocardiogram, the risk is decreased. For patients with a compromised cardiac condition, pegylated liposomal doxorubicin and dexrazoxane may minimize anthracycline-induced functional damage. Relapse is another problem. For patients with high-risk IPI scores, lenalidomide is an option[15,25,26], but no patient in our study received this agent. Only one patient in our study received rituximab maintenance therapy. This patient has experienced over 5 years of disease-free survival. Rituximab, which is the CD20 monoclonal antibody, may have the potential to erase minimal residual tumor cell burden after intensive induction chemotherapy. It can prolong the PFS in advanced-stage follicular lymphoma, but in aggressive lymphomas such as DLBCL, rituximab maintenance is controversial [27]. Furthermore, studies have shown that males may need higher doses of rituximab to improve its efficacy [28].

EBV infection, like c-Myc rearrangement, was once thought of as a signal of Burkitt’s lymphoma; currently, both factors have been found to be positive in DLBCL. The exact role of EBV in DLBCL is largely unexplored [29]. More research is needed to identify this molecular marker, to expand this entity and to improve its prognosis[30,31].

## Conclusion

EBV-positive DLBCL in the elderly is a rare malignancy with a very poor prognosis. This disease is associated with adverse clinical outcomes and pathologic characteristics. In this study, EBV-positive DLBCL in the elderly had inferior treatment responses and survival outcomes compared with EBV-negative cases. The role of EBV in EBV-positive DLBCL in the elderly and the optimal management for this subgroup warrant further investigation.
